# Swarming Behavior in Plant Roots

**DOI:** 10.1371/journal.pone.0029759

**Published:** 2012-01-17

**Authors:** Marzena Ciszak, Diego Comparini, Barbara Mazzolai, Frantisek Baluska, F. Tito Arecchi, Tamás Vicsek, Stefano Mancuso

**Affiliations:** 1 CNR-Istituto Nazionale di Ottica, Florence, Italy; 2 LINV-Department of Plant Soil & Environmental Science, University of Florence, Florence, Italy; 3 Center for Micro-BioRobotics, Istituto Italiano di Tecnologia, Pontedera (PI), Italy; 4 Institute of Cellular and Molecular Botany, University of Bonn, Bonn, Germany; 5 Department of Physics, University of Florence, Florence, Italy; 6 Department of Biological Physics, Eötvös Loránd University, Budapest, Hungary; Tel Aviv University, Israel

## Abstract

Interactions between individuals that are guided by simple rules can generate swarming behavior. Swarming behavior has been observed in many groups of organisms, including humans, and recent research has revealed that plants also demonstrate social behavior based on mutual interaction with other individuals. However, this behavior has not previously been analyzed in the context of swarming. Here, we show that roots can be influenced by their neighbors to induce a tendency to align the directions of their growth. In the apparently noisy patterns formed by growing roots, episodic alignments are observed as the roots grow close to each other. These events are incompatible with the statistics of purely random growth. We present experimental results and a theoretical model that describes the growth of maize roots in terms of swarming.

## Introduction

To exploit soil resources optimally, plants have developed intricate root systems that are characterized by complex patterns and based on the coordinated group behavior of the growing root apices [1]–[3]. The communication channels among different root apices of the same plant depend on both fast electrical [4], [5] and slower hydraulic and chemical signals [6], [7]. In addition, secreted chemicals and released volatiles allow rapid communication among individual roots of the same plant and among roots of different plants. It has been shown that plants can distinguish between self and non-self roots [8]–[11] and that the sensory information collected by one plant is shared with neighboring plants [12], [13] to optimize territorial activities [10]–[13], including competitive behaviors [14] and symbioses with fungi and bacteria [1], [2], [6], [7]. Growing roots are well known to generate electric fields around their extending apices where their magnitude is maximum. Such fields are changing when roots are subject to gravitational stimuli [15] and are tightly linked with the polar transport of auxin, which controls root growth, tropism and root navigation [16]. Moreover, they affect protein distributions, conformations and activities and play an important role in regulating endocytosis and cytoskeleton [17]. On the other hand, it has been shown that external electric fields influence growing roots [18], for instance through the electrotropism phenomenon [19]–[21]. Recently, these evidences have led to the hypothesis that swarm intelligence might not be restricted to animals and that other complex systems involving mutual interactions, such as plant roots, could also be accurately described in terms of swarming [22]. Swarming refers to a situation in which individuals of a group of animals create a spatiotemporal order characterized by the alignment of directions and maintenance of equal speeds and distances. The emergence of swarming has been observed in many biological systems, such as fishes [23], bees [24], birds [25], bacteria [26] and insects [27]–[29], and in many human activities, including the correlated movements of pedestrians [30] and traffic [31] (for a recent review of swarm intelligence in animals and humans, see [32]). Such order may emerge from simple rules based on mostly local protocols [33]. One approach to modelling collective motion in biological systems uses self-propelled particles, i.e., particles that make decisions according to certain rules. A simple model proposed by Vicsek et al. [34] to describe such particles focused mainly on the emergence of directional alignment. Further studies have considered phase transitions in collective motions [35], adaptive velocities models [36] and network topologies [31]. A more biologically realistic approach to modelling swarming behavior considers local repulsion, alignment and attractive tendencies based on the relative positions and orientations of individuals [25], [37], [38]. Recent studies [39]–[41] have demonstrated that by considering only three laws of repulsion, attraction and heading alignment, it is possible to generate behavioral patterns very similar to those of real swarms. Thus far, questions of plant behavior have never been considered in terms of swarming. Here, we present experiments that demonstrate the existence of guided interaction in plant groups, providing evidence for swarming behavior in growing roots. Although chemical substances could play a role in the observed swarm-like root behavior, the ability of root apices of generating and detecting electric fields represents a further and more plausible mechanism. In addition, we propose a theoretical model based on the dynamics of self-propelled particles affected by forces of repulsion and attraction that depend on the positions and orientations of individuals growing in a given area. To distinguish observed root patterns from random root growth, we used an algorithm to generate random growth, which we compare to the experimental data.

## Results

### Characteristics of growing roots

**Figure 1:**
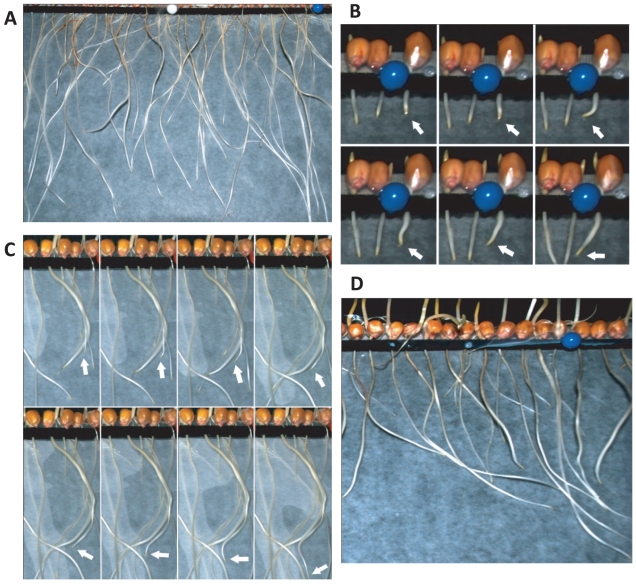
Experimental growth of maize roots. A. An image showing root growth in one of 10 experiments. B. Decision-making by the root apex: the movement of the root in the two opposite directions before committing to a growth direction. C. The alignment of one root with others based on distance, i.e., without physically contacting neighbors. D. An example of collective behavior: a group of roots chooses the same growth direction.

Fig. 1 A shows the growth of maize roots over time, displaying the roots' complex trajectories. The separation between the seeds is approximately 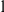 cm. The direction of growth is controlled by the root apex, as shown in Fig. 1 B (see also Video S1 and S2). There are two types of interaction between the roots: alignments based on distance and repulsions. The crossing of roots is also occasionally observed, which could be interpreted as attraction followed by repulsion or as no interaction. The adjustment of the direction of growth (alignment) is often based on distance, as shown in Fig. 1 C. This dependence of growth on mutual distance indicates the existence of a form of signaling, possibly mediated by chemical substances. Another important feature of the growing roots is their collective behavior. In Fig. 1 D, a group of roots are shown to have chosen the same direction of growth, forming a type of cluster. It should be noted that water was homogenously distributed, and the experimental setup was well protected from the external environment to avoid localized stimuli.

### The random-growth model

We generated random growing paths by considering an assembly of independent particles separated initially by distance 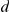 with associated velocity vectors; there were no forces from the neighbors. At each time step, the particle positions are updated according to 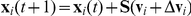, where we considered a unitary time step. The velocities 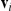 have absolute value 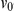, and 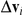 is a stochastic term consisting of random numbers distributed uniformly in the interval 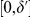. The direction of growth is determined by the matrix 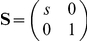, where 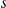 is a two-valued variable where the values 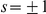 are selected randomly with probability 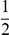 (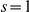 for growth to the right and 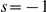 for growth to the left). The introduction of 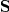 to the model was made in order to direct the growth in a downward direction (more precisely, to restrict movements to the east-south and west-south directions).

### Model of non-random growth

**Figure 2:**
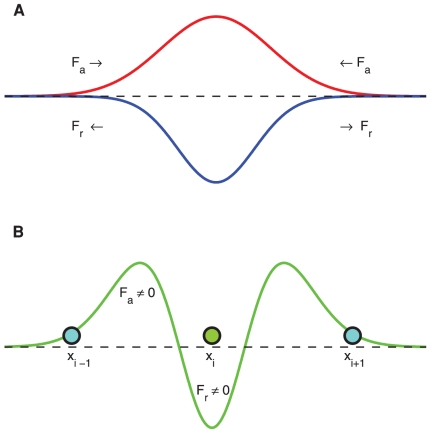
Forces acting on the roots. A. The force of attraction, 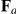, and the force of repulsion, 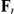. B. The effective force associated with each root being a sum of profiles shown in A. Filled circles indicate the position of roots.

In the model of non-random growth, we consider the root apex or tip as a moving particle, and we treat the length of the root as the temporal history of the particle. Each particle is described by a velocity vector, the orientation of which denotes the direction in which the root apex moves. We assume that each seed produces only one main root and no collateral roots. This simplification is reasonable because the main root provides the longest and clearest history of interaction with its neighbors. The roots can interact with their neighbors at any point along their lengths. We assume that each root grows at a slightly different speed, giving rise to different root lengths. While this feature is clearly observed in experiments, thus motivating its inclusion in the theoretical model, we remark that this is not crucial in determining the growing-roots patterns. We have introduced the variation in speeds in accordance to experimental data which revealed that the variability in root lengths is always observed. Moreover, each plant is assumed to interact with neighboring plants causing spatial attraction or repulsion depending on the individual root responses. As mentioned before electric fields may be a possible mechanism of such interaction. These attractive and repulsive forces become effective when a certain distance separates the roots and leads them to grow closer or far away to together. As two roots approach each other, the mechanisms of direction adjustment (alignment) would switch on. The root apices adjust their direction as they detect their neighbors within a certain radius 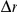. The roots are constrained to grow on a two-dimensional surface, so no-flux boundary conditions are considered. The time evolution of each root apex's vector marks the growing path of the root on the surface. The simulations were performed in a rectangular cell of horizontal length 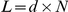, where 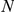 is the number of seeds and 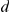 is the spatial separation between two adjacent seeds. The vertical size is marked by the longest root, 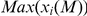, where 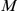 is the last time value. The spatiotemporal history of the moving particle marks the shape of the whole root. The position of the 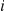
th root apex is updated according to
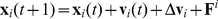
(1)where we considered a unitary time step. Because roots grow at slightly different speeds, instead of an equal velocity 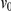 for all particles, we consider different velocities for each root. More precisely, the velocities 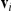 have the absolute value 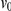, and 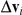 is the fluctuation term composed of random numbers distributed uniformly in the interval 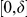. The angle 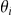 with respect to the horizontal direction is updated according to 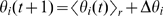, where 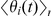 denotes the average direction of the growing root apices within a radius 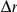 surrounding root apex 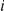. The average direction is defined as 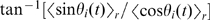. Small noisy fluctuations 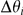 in the angle are considered to incorporate the effects of varying environmental conditions on the roots' decision-making. The fluctuations are modelled as random numbers chosen with a uniform probability from the interval 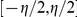. The forces produced by the root 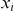 that act on the neighbors are schematically presented in Fig. 2. We assume a Gaussian dependence of the force magnitude as a function of the distance, so they become stronger as the distance between the roots diminishes. The attractive and repulsive forces produced by the root 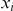 and acting on the root 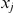 (corresponding to the profiles in Fig. 2 A) are defined as:
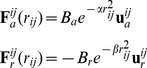
(2)where parameters 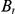, 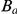, 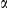 and 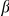 are constant, with additional constraint 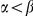. The total force acting on the root 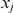, which models the root decision making on how to interact with a given neighbor, is then 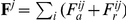. In Fig. 2 B we show that the force generated by the root situated in between the two neighbors defines two regions, i) where only the repulsive force is present and ii) where only the attractive force is present. When more roots are situated in a certain spatial region they all contribute in determining the resultant force acting on a particular neighboring root. For the sake of simplicity, we assume that the forces 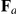 and 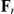 act only in the horizontal directions. The action of the gravitational force is modelled by assuming that when the critical value 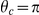 of the angle is reached, the root apex starts to adjust its direction according to 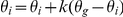, where 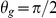 and 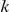 is a constant parameter. In other words, the root apex is pushed to align with the direction of the gravitational force. The growing paths of the roots are adequately modelled by adjusting the system parameters.

### Data analysis from the experiments, the non-random model and the model of random growth

**Figure 3:**
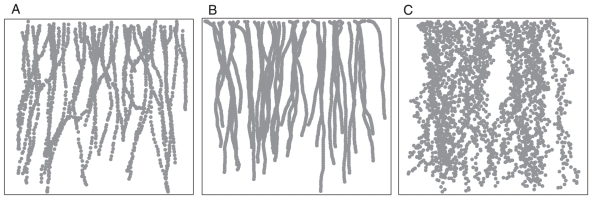
Growing root paths marked by data from the: A. experiment; B. non-random model; and C. model of random growth. The parameter values used in B are: 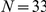, 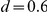, 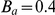, 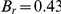, 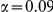, 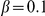, 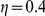, 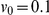, 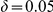, 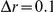 and 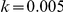. The parameter values used in C are: 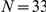, 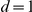, 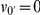 and 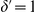.

**Figure 4:**
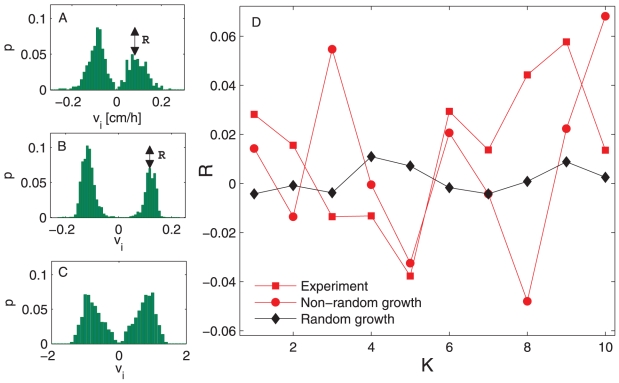
The binomial distributions and the indicator of direction of the growing roots 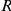
 **.** The sample bimodal distribution calculated from the: A. experiment; B. non-random model; and C. model of random growth. Double arrows mark the difference between maxima of bimodal distributions. This difference defines the value of 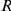. D. The indicator of direction of the growing roots, 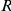, exhibits high variations in the case of non-random growth, both in experiments and numerical simulations, and low variations in the case of random-growth model. The parameter values used in B and C are as in Fig. 3 B and C, respectively. 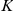 stands for experiment number.

We calculate the velocity vectors associated with root growth rate and direction for 10 experiments and analogously for 10 realizations of the numerical experiments. In numerical simulations, approximately the same number of seeds and temporal data points are considered as in the experiment. The sample growing paths from the experiment, the model of non-random growth and the model of random growth are shown in Fig. 3 A, B and C, respectively. The probability distributions of the velocities are bimodal, revealing the growth direction: negative velocities denote roots growing to the right, and positive velocities denote roots growing to the left (all roots also grew downward). In the case of non-random growing (both in the model and experiment) the distributions tend to be asymmetric (see Fig. 4 A and B, respectively), meanwhile in the case of random growing the bimodal distribution is symmetric (see Fig. 4 C). We define a symmetry indicator of the binomial distribution 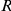, as the difference 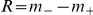, where 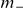 and 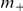 are the local maxima of the left- and right-hand sides of the distribution, respectively. When 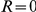, as is the case of a random-growth model, this corresponds to zero mean velocity vector (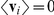). On the other side, when 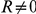, the corresponding mean velocities have non zero values (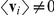). We plotted the indicator 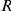 for the results from the experiment and the models of non-random and random growth in Fig. 4 D. The observed asymmetries in distributions, with 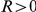 or 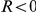 for various experiments, give an indication of episodic alignments of the directions of neighboring root apices. When plotting the mean probabilities 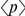 for all experiments, we notice large fluctuations in the heights of these distributions (Fig. S1 A). The same phenomenon is observed in the model based on attractive and repulsive forces (Fig. S1 B). In contrast, in the random-growth model, we observe homogenous distributions for all directions and small fluctuations in the distribution heights (Fig. S1 C). The consequences of such asymmetry are also observed in the distributions of the horizontal distances of the root apices from their seeds, defined as 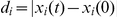. We report the probability distributions of these distances and their means in Fig. S2. The mean values of the probabilities of 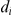 are associated with higher standard deviations in the experiment (Fig. S2 A) and the model of non-random growth (Fig. S2 B), whereas the probabilities remain fixed and have relatively small standard deviations in the model of random growth (Fig. S2 C). This pattern indicates that the root apices grow in response to their neighbors rather than randomly.

### Indicator for velocity correlations

**Figure 5:**
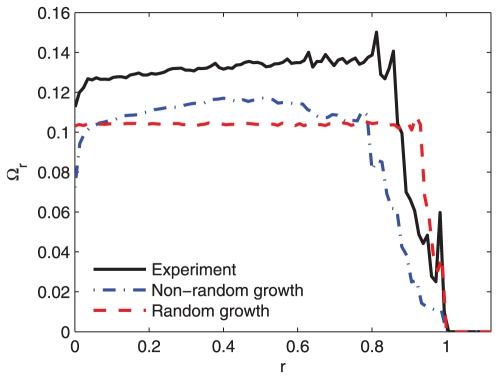
Velocity correlation indicator 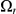
 **of the experiment (solid line), model of non-random growth (dotted-dashed line) and model of random growth (dashed line).** The value of radius 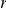 was normalized in all cases to equal 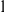 at the system size. The curve obtained from a random growth model differs essentially from the curves obtained from experiment and the non-random growth model. The parameter values used in numerical simulations of non-random growth model are as in Fig. 3 B, meanwhile for the random growth model are: 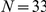, 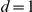, 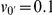 and 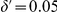.

As described in the previous paragraph, the asymmetry in the heights of the bimodal distributions may indicate the existence of a preferred direction of growth or episodic alignments of directions between neighboring roots. To investigate, we calculated the spatial correlations between velocity vectors as a function of the distances between the roots. More precisely, we defined a radius 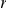 and the radial area from 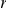 to 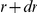 and then calculated the mean of the absolute value of the velocity differences in the circular area enclosed in the interval 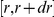, 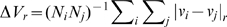, where 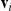 is the velocity of the reference particle (all points along the root body are considered) and 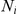 is the total number of the reference particles. 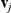 and 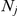 describe the velocities of all particles within the radius 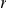 and their total number, respectively. We then calculated the mean absolute velocity differences of the particles which could be found within a given radius 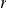 around the reference particle 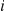, 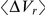, over all experiments and normalized it by the overall mean velocity 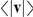, which gave a dimensionless description of the velocity correlations, defined as follows:
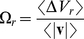
(3)This dimensionless indicator was used to compare the correlations in directions of growing in the experiment and the numerical simulations of random and non-random growth. The indicator 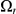 was calculated for all three cases, and its dependence on 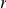 plotted in Fig. 5. The curves from the experiment (solid line) and the non-random growth model (dotted-dashed line) exhibit a positive slope, indicating the presence of correlations in the velocities at small values of 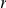. For the random-growth model (dashed line), the slope is equal to zero; the particle motion is random and does not depend on the velocities of its neighbors.

## Discussion

In the non-random model, the mean probability distribution of the velocities tends very slowly to a symmetric shape, and large fluctuations in the differences between the heights of the bimodal peaks are observed between individual experiments. In the random-growth model, different patterns are observed. The effect of noise makes the distributions symmetrical, and consequently, the fluctuations in the differences of the heights of the bimodal distributions are smaller. However, when interactive forces are present, the distributions become asymmetric and the fluctuations larger. These fluctuations arise from the alignment of growing roots and some type of synchronization of growth directions. The results of the experiment and non-random model differ qualitatively from those of the random-growth model, and the experimental results differ slightly from the predictions of the non-random model. This difference may be related to the fact that the non-random model considers only a few of the most essential ingredients that contribute to non-random growth. Other elements which have been neglected for the sake of simplicity contribute to root interactions and behavior: for instance, inhomogeneities in water distribution, initial distances between the seeds, or angle of the support on which the roots are growing. Despite these facts the proposed model allowed us to qualitatively reproduce the observed growing root patterns by making use of few basic ingredients.

Concerning the swarming behavior in roots, one of the advantages could be the efficient chemical modification of the soil in their vicinity. This would allow the maintenance of specific root micro-niche which is optimal for their physiological performances, in particular, the extraction of the essential nutrients from the soil and the defense against pathogens.

In conclusion, the experiments revealed that the qualitative features of root growth are well explained by a model of swarm behavior. The main insight gained in this study is that the root apices act as decision-making centers, giving rise to correlations in the growth patterns. We have identified a few key ingredients allowing us to explain and reproduce qualitatively the observed phenomenology, in particular, the angle adjustment and the attractive and repulsive interactions. Repulsive forces have been considered in the description of the polymer brushes [42] displaying similar patterns. However, in this case the brushes never intersect and the alignments are absent. In contrast, roots display both alignments and intersections between them, thus motivating the further inclusion in our model of the attractive force and angle adjustment.

## Materials and Methods

Maize seeds were germinated at 24
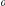
C and allowed to grow for 4 days before being used in the experiments. The seeds were placed in a dark room on top of a plain supporting table fixed at approximately 75 degrees from horizontal. Water was homogenously distributed at the bottom of the supporting table and wicked up by the paper covering the table. Cameras were programmed to take photos every 30 minutes for 7 days on average. Images were processed with Tracker 4.0 software from the Open Source Physics collection, and data were processed by using routines written in MATLAB.

## Supporting Information

Figure S1
**Probability distribution of the velocities of growing roots.** A. Experiment, B. non-random growth model and C. random-growth model. The parameter values used in B are: 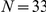, 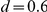, 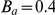, 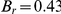, 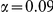, 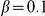, 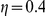, 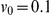, 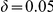, 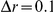 and 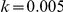. The parameter values used in C are: 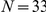, 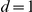, 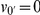 and 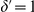. The three panels from the left show the representative distributions observed in single experiments and numerical realizations. The right panels show the averaged probability distributions.(EPS)

Figure S2
**Probability distributions of the distances of the root apices from their seeds:** 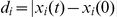 **.** A. Experiment, B. non-random growth model and C. random-growth model. The parameter values in B and C are as in Fig. S1. The three panels from the left show the representative distributions observed in single experiments and numerical realizations. The right panels show the averaged probability distributions.(EPS)

Video S1 **Experiment with a growing group of maize roots.**
(MOV)

Video S2 **Experiment with a single growing maize root.**
(MOV)
