# Correction: Swarming Behavior in Plant Roots

**DOI:** 10.1371/annotation/8e6864fc-c4b7-46e7-92b3-80767f4a5d3a

**Published:** 2012-03-07

**Authors:** Marzena Ciszak, Diego Comparini, Barbara Mazzolai, Frantisek Baluska, F. Tito Arecchi, Tamás Vicsek, Stefano Mancuso

There was an error in the funding statement. The correct funding statement is: "This work was supported by Marie Curie European Reintegration Grant N. 239324 MIPAN (Memory and information processing in assemblies of neurons) within the 7th European Community Framework Program, http://cordis.europa.eu/fp7/dc/index.cfm [^] (MC), Ente Cassa di Risparmio di Firenze, URL:http://www.entecarifirenze.it/ [^] (SM) and EU ERC Project N. 227878 COLLMOT (Complex structure and dynamics of collective motion), http://erc.europa.eu/ [^] (TV). The funders had no role in study design, data collection and analysis, decision to publish, or preparation of the manuscript." 

